# The Association Between Health Information Seeking on the Internet and Physician Visits (The Seventh Tromsø Study - Part 4): Population-Based Questionnaire Study

**DOI:** 10.2196/13120

**Published:** 2020-03-05

**Authors:** Kassaye Yitbarek Yigzaw, Rolf Wynn, Luis Marco-Ruiz, Andrius Budrionis, Sunday Oluwafemi Oyeyemi, Asbjørn Johansen Fagerlund, Johan Gustav Bellika

**Affiliations:** 1 Norwegian Centre for E-health Research University Hospital of North Norway Tromsø Norway; 2 Department of Clinical Medicine Faculty of Health Sciences UiT The Arctic University of Norway Tromsø Norway; 3 Division of Mental Health and Addictions University Hospital of North Norway Tromsø Norway; 4 Department of Community Medicine Faculty of Health Sciences UiT The Arctic University of Norway Tromsø Norway

**Keywords:** eHealth, internet, health care service, physician visit, Tromsø study, health information seeking, Web search engine, health app, social media, video search engine

## Abstract

**Background:**

The internet is being widely used for seeking health information. However, there is no consensus on the association between health information seeking on the internet and the use of health care services.

**Objective:**

We examined the association between health information seeking via the internet and physician visits. In addition, we investigated the association between online health information seeking and the decisions to visit and not to visit a physician.

**Methods:**

We used the cross-sectional electronic health (eHealth) data of 18,197 participants from the seventh survey of the Tromsø Study (Tromsø 7). The participants were aged ≥40 years and living in Tromsø, Norway. We used logistic regression models to examine the association between online health information seeking and physician visits, the decision to visit a physician, and the decision not to visit a physician, with adjustment for the demographic status, socioeconomic status, and health status of the participants.

**Results:**

The use of Web search engines was associated with a physician visit. However, the association was moderated by age, and the OR decreased as age increased. The ORs for the use of Web search engines were 1.99 (95% CI 1.94-2.02) and 1.07 (95% CI 1.03-1.12) at ages 40 and 80 years, respectively. The decision to visit a physician was associated with the use of Web search engines (OR 2.95, 95% CI 2.03-4.46), video search engines (OR 1.43, 95% CI 1.21-1.70), and health apps (OR 1.26, 95% CI 1.13-1.42). The association between social media use and the decision to visit a physician was moderated by gender. Women who used social media had 1.42 (95% CI 1.31-1.55) times higher odds of deciding to visit a physician, whereas the decision to visit a physician was not different between men who used social media and those who did not use social media. Conversely, the decision not to visit a physician was associated with the use of Web search engines (OR 2.78, 95% CI 1.92-4.18), video search engines (OR 1.27, 95% CI 1.07-1.51), social media (OR 1.28, 95% CI 1.10-1.49), and health apps (OR 1.20, 95% CI 1.07-1.35).

**Conclusions:**

Health information found on the internet was positively associated with both the decision to visit a physician and the decision not to visit a physician. However, the association of health information seeking with the decision to visit a physician was slightly stronger than the association with the decision not to visit a physician. This could imply that the use of eHealth services is associated with a resultant increase in physician visits. In summary, our findings suggest that the internet serves as a supplement to health care services rather than as a replacement.

## Introduction

Access to the internet is currently widespread. In 2017, around the time the data for this study were collected, the internet coverage of Norwegian households was 97%. Internet access varies between 91% and 100% depending on household income. Ninety percent of Norwegians aged between 16 and 79 years use the internet on a daily basis, with similar internet usage between women (87%) and men (90%) [1].

Broad access to the internet has triggered a rapid growth in the use of the internet for health-related applications, such as accessing and transferring health information and receiving guidance and support [2-6]. Health information seeking is considered the most common and influential use of the internet [7-11]. Among Norwegian internet users, 62% used the internet for seeking health information, with a higher proportion of women and younger adults [1], which is consistent with the findings in other countries [8,12-14].

The perceived benefits of online health information seeking include widespread access to health information, convenience (ie, ease and speed), and anonymity [15-19]. These benefits are expected to enable individuals to play an active role in their health care, make better-informed decisions, and possibly improve health outcomes [18,20,21]. There are also concerns that the variable quality of online health information combined with limited ability to critically evaluate health information may contribute to negative outcomes, such as unnecessary physician visits, delays in seeking necessary medical care, change in treatments, and seeking alternative treatments that can be harmful [5,16,17,22-26].

In Nordic countries, including Norway, and many other European countries, including the United Kingdom, general practitioners (GPs) represent the basis of publicly funded health services. GPs typically have lists of patients who they are responsible for and see more or less regularly. GPs are expected to diagnose and treat a major proportion of patients and refer only those who need more specialized health services to hospitals for further assessment and treatment (ie, the “gatekeeper” function) [27]. The threshold to seek consultation with a GP may be relatively low, but many GPs are very busy and patients nevertheless have to decide whether their current problem warrants a physician visit [27].

Searching for health information is a complex process influenced by a range of factors. Lambert & Loiselle [28], in their review, found that the behavior of health information searching is often studied within the context of coping with illness, involvement in medical decision-making, or preventive behavior. Different theoretical frameworks have been applied in research on health information searching [28]. The framework of Miller [29] is one of the most cited frameworks, and a differentiation is made between those who actively engage with information (monitoring) and those who avoid such information (blunting) [30]. Psychological factors, including cognitive and emotional factors [31,32], might be central in the decision-making process for many health information searchers. The process of online health information searching may be powered by not only a need for information about some health- or illness-related topic but also different emotional states, such as anxiety [33], and people with health anxiety are more likely to search for health information [33]. Moreover, how people react to the health information they find online may also vary according to a range of factors, including emotional factors [31,32]. Although finding the required information can result in a positive emotional reaction, it may also result in confusion or increased anxiety [34]. Similarly, the decision to visit a physician or not to visit a physician after finding health information online is a complex process that could be influenced by many different factors, some of which will be examined in this study.

Health information seeking is known to highly influence subsequent health-related behavioral decisions. It has been reported that 46% of European adults [8] and 48% of American adults [4] who sought health information via the internet used the information they found to decide whether they needed to see a physician.

Prior studies have found different results regarding the question about whether online health information searching impacts physician visits. Some studies have suggested that searching for online health information can be conducive to increased physician visits. A proposed mechanism underlying this increase in visits is that some individuals may find it difficult to interpret complex and uncertain medical knowledge without the help of health care professionals [35-41]. Therefore, more access to health information via the internet may lead to more uncertainties in understanding health conditions and, consequently, to additional contact with health care professionals. Other studies have found that online health information searching reduces traditional health care service consumption [42,43], and some studies have found no such association [44]. In this study, the objective was to make an accurate assessment of the association between health information seeking and health care service use. We believe that this will be useful for estimating future health care service need in Norway and other countries and for informing future policies related to health care in general and electronic health (eHealth) services in particular.

Studies often capture the outcomes of health information seeking in terms of the decision to visit a physician as a single question that asks if the health information individuals found online led them to decide to visit a physician or not [40]. However, some studies had two questions that capture both outcomes [44], as a single question may not capture both outcomes, given that individuals may make both decisions at different time points in a study period. Therefore, a separate question is needed for each of the outcomes.

The seventh survey of the Tromsø Study (Tromsø 7) included a questionnaire with a wide variety of questions, such as those on the use of eHealth tools, use of health care services, socioeconomic status, and health status. We explored a subset of the data from this questionnaire in a series of four papers. In paper 1, we presented the main findings regarding the characteristics of the participants and their use of eHealth [45]. In paper 2, we studied how the presence of different illnesses influences the use of eHealth [46]. In paper 3, we examined the psychological and emotional outcomes with the use of eHealth [34]. Paper 4 (this paper) aims to study the association between health information seeking using eHealth tools and physician visits. This paper also investigates the associations of online health information seeking with decisions to visit and not to visit a physician.

## Methods

### The Study Site

The participants of the study are inhabitants of Tromsø municipality. Tromsø is a major city in north Norway with a population of around 75,000 inhabitants. The city of Tromsø is located in the sub-Arctic region at 69 degrees North. North Norway is a sparsely populated area with most inhabitants working within the public sector, including health care, education, and administration, and the service sector. Other important employment areas in north Norway are tourism, fishery, agriculture, and some industries.

### The Tromsø Study

The Tromsø Study is a population-based longitudinal health study conducted by UiT The Arctic University of Norway, the National Health Screening Service, and others [47]. Tromsø 7 was conducted in 2015-2016 with a focus on inhabitants aged 40 years or above. However, this is the first time the Tromsø Study collected eHealth related information. Therefore, this paper, which is based on eHealth survey data, involves a cross-sectional study. A total of 21,083 individuals (11,074 women and 10,009 men) participated in the study, with a response rate of 65%.

### Independent Variables

The variables used in this study were measures of health information seeking via Web search engines (ie, Google), video search engines (ie, YouTube), social media (ie, Facebook), and smartphone or tablet health apps. The variables were constructed from four questions regarding whether a participant had used each of the eHealth tools in the last year. The responses to the questions were “never,” “once,” “sometimes,” and “often.” We subsequently dichotomized the responses into “never” and “ever,” where “ever” includes once, sometimes, and often.

The other independent variable was a dichotomous measure of whether a participant reported having one or more diseases in the last year. The variable was constructed from questions regarding whether a participant had diseases, such as high blood pressure, heart attack, heart failure, atrial fibrillation, angina, stroke, diabetes, kidney disease, bronchitis, asthma, cancer, rheumatoid arthritis, arthrosis, migraine, psychological problems, and chronic pain. The responses to the questions were “no,” “yes,” and “yes, previously.”

Age, gender, education, household income, occupation, and self-reported health, which are known to influence health information-seeking behaviors, were also controlled for in this study. Age was converted into 10-year intervals, as a small age difference is not associated with a large change in the outcome variables.

### Dependent Variables

In the Tromsø Study, there were three questions examining different aspects of health service use. The first dependent variable was a dichotomous measure of whether the participant had a physician visit in the last year. The variable was constructed from questions that asked whether a person visited a GP, emergency care practitioner, psychologist, or psychiatrist in the last year.

The second dependent variable measures the decision to visit a physician following health information seeking on the internet. The variable was constructed from a question that asked whether a participant decided to visit a physician according to the information found on the internet. The response to the question was “never,” “once,” “sometimes,” or “often.” We dichotomized the responses into “never” and “ever,” where “ever” includes once, sometimes, and often.

The third dependent variable measures the decision not to visit a physician following health information seeking on the internet. The variable was constructed from a question that asked whether a participant decided not to visit a physician according to the information found on the internet. The response to the question was “never,” “once,” “sometimes,” or “often.” We dichotomized the responses into “never” and “ever,” where “ever” includes once, sometimes, and often.

The physician visit variable measures whether a participant visited a physician in the last year. It is known that people seek health information for a wide variety of purposes, including deciding whether to visit a physician, preparing for a physician appointment, and reassurance, second opinion, and expanding knowledge on the information received from health care providers after a physician visit [15,16,18,48]. In other words, a participant may seek health information before or after a physician visit. Even when a participant seeks health information before a physician visit, the health information the participant obtains may not necessarily be used for deciding whether to visit a physician.

The variable of the decision to visit a physician measures whether a participant decided to visit a physician because of the health information the participant read online. Similarly, the variable of the decision not to visit a physician solely measures whether a participant decided not to visit a physician because of the health information the participant read online. Therefore, a subset of actual physician visits can be associated with decisions to visit made after reading online health information.

If a participant never decides to visit a physician according to health information read on the internet, it does not necessarily mean the participant used the information to decide not to visit a physician and vice versa. The participant may have sought the health information for other purposes, may not have found the information useful, or may not have understood the information well enough to base decisions on it.

### Study Sample

**Figure 1:**
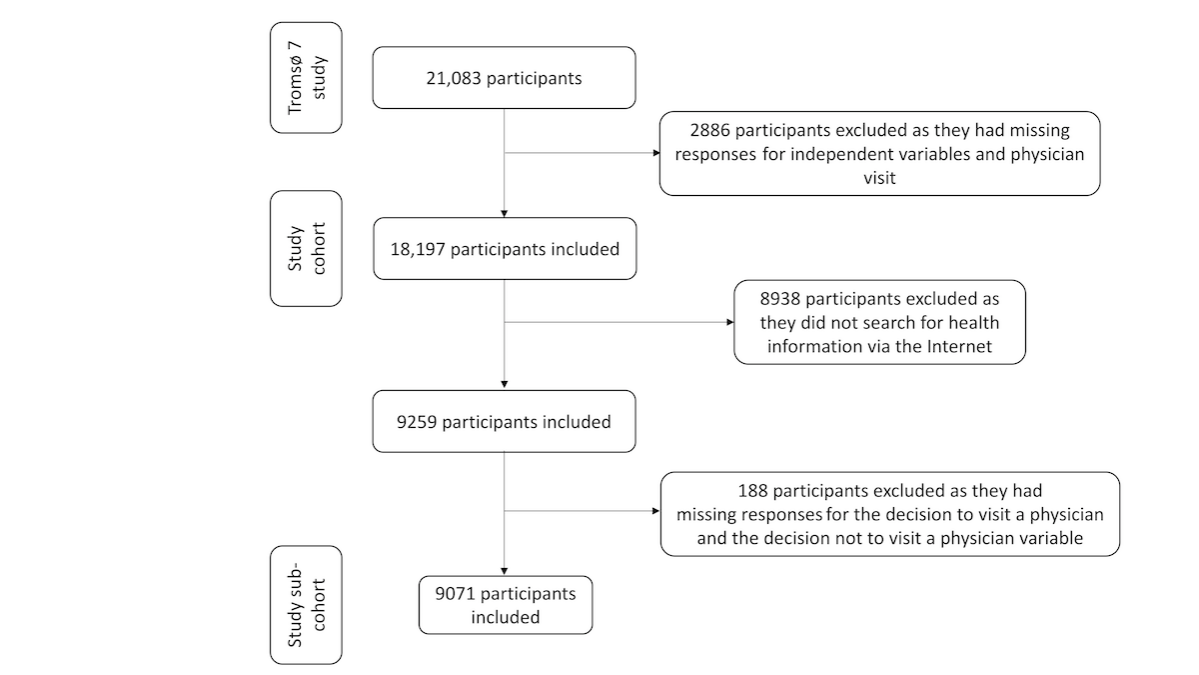
The study sample selection workflow.

Figure 1 shows the study sample selection workflow. Of the 21,083 participants in Tromsø 7, we excluded 2886 participants who had missing responses for one or more of the variables, such as age, gender, education, household income, occupation, Web search engine use, video search engine use, social media use, health app use, diseases, self-reported health status, and physician visit. The final study sample consisted of 18,197 participants (9251 women and 8946 men).

Of the 18,197 participants included in this study, 9259 participants sought health information via one or more eHealth tools. Of these 9259 participants, we subsequently excluded 188 participants with missing information regarding the variables of the decisions to visit and not to visit a physician. As a result, a subcohort that consisted of 9071 participants (5110 women and 3961 men) was created.

### Statistical Analysis

Descriptive statistics were calculated to summarize the dependent and independent variables. Multivariable logistic regression models were fitted for the following three outcome variables of interest: (1) physician visit; (2) decision to visit a physician according to health information read on the internet; and (3) decision not to visit a physician according to health information read on the internet. The first model was fitted on the whole cohort dataset (n=18,197), and the other two models were fitted on the subcohort dataset (n=9071).

We used purposeful selection of independent variables for each of the logistic regression models as proposed by Hosmer et al [49]. First, we fitted the model with only one independent variable. Second, we fitted a multivariable model with all independent variables whose *P* values were <.25 in the previous step. Third, we iteratively checked whether variables not significant (Wald test and .05 alpha value) in the multivariable model provided important adjustment to other variables that remained in the model. Important adjustment was measured by a change in coefficients by more than 20% between the multivariable models with and without the variable. Variables that provided important adjustment were added back into the multivariable model. Fourth, to ensure we did not miss any important variables, we added independent variables not added in the second step, one at a time to the multivariable model, using the Wald test to verify the effect of each additional variable at an alpha value of .05. Thereafter, we added all independent variables that were significant to the multivariable model. Fifth, we explored possible interactions of age, gender, and education with the use of eHealth tools. We also explored possible interactions between age and disease, age and self-reported health, age and household income, education and household income, education and disease, education and self-reported health, household income and disease, self-reported health and disease, and household income and self-reported health. We added the interactions, one at a time, to the multivariable model at the end of step four and tested significance using a likelihood ratio test and an alpha value of .1. Following separate analysis of each of the interaction terms, we added each significant interaction term to the model at the end of step four and removed interaction terms that were not significant with the Wald test and an alpha value of .05. Thereafter, we fitted the model excluding the nonsignificant interactions. Sixth, we checked the fitness of the model with regard to the data using the Hosmer-Lemeshow goodness-of-fit test.

We tested statistical significance at an alpha level of .05. All analyses were conducted using R statistical software (version 3.4.0; R Project for Statistical Computing, Vienna, Austria). All the ORs reported in the paper are adjusted ORs.

### Ethics

Tromsø 7 was approved by the North Norway Regional Committee for Medical and Health Research Ethics (REK Nord, reference 2014/940). All participants provided written informed consent for Tromsø 7.

## Results

### Participants' Characteristics

Characteristics of the study participants are summarized in Table 1. Of the 18,197 study participants, 9251 (50.84%) were women and 8948 (49.16%) were men. The mean age of the overall study sample (n=18,197) was 56.38 years, with 51.10% (9298) of participants having a college education. About three-fourths of the participants (73.22%, 13,323/18,197) had one or more diseases, 50.88% (9259/18,197) sought health information via one or more eHealth tools, and 80.38% (14,627/18,197) had a physician visit in the last year.

**Table 1:** Characteristics of the study sample.

Variable		Cohort (n=18,197)		Subcohort (n=9071)	
		Count	Percentage	Count	Percentage
**Gender**					
	Female	9251	50.84	5110	56.33
	Male	8946	49.16	3961	43.67
Age, years		56.38^a^	11.00^b^	52.60^a^	9.25^b^
**Education**					
	Primary or secondary	3831	21.05	970	10.69
	Upper secondary	5068	27.85	2280	25.13
	Less than 4 years of college	3650	20.06	2086	23.00
	Four years or more of college	5648	31.04	3735	41.18
**Household income (US)^c^**					
	<39 000 US	2132	11.72	568	6.26
	39 000 – 61 000 US	3725	20.47	1632	18.00
	61 000 – 83 000 US	3242	17.81	1519	16.74
	83 000 – 111 000 US	4396	24.16	2449	27.00
	>111 000 US	4702	25.84	2903	32.00
**Occupation**					
	Full-time work	11,145	61.25	6461	71.23
	Part-time work	1453	7.99	783	8.63
	Unemployed	122	0.67	70	0.77
	Housekeeping	99	0.54	29	0.32
	Retired	3720	20.44	912	10.05
	Student/military service	55	0.30	39	0.43
	Disability and other family welfare benefits	1603	8.81	777	8.57
**Disease**					
	Yes	13,323	73.22	6688	73.73
	No	4874	26.78	2383	26.27
**Self-reported health status**					
	Very bad	54	0.29	27	0.30
	Bad	909	4.99	508	5.60
	Neither bad nor good	4583	25.19	2082	22.95
	Good	9891	54.36	5003	55.15
	Very good	2760	15.17	1451	16.00
**Physician visit**					
	Yes	14,627	80.38	7496	82.64
	No	3570	19.62	1575	17.36
**Web search engine use**					
	Ever	8956	49.22	8773	96.71
	Never	9241	50.78	298	3.29
**Video search engine use**					
	Ever	900	4.95	875	9.65
	Never	17,297	95.05	8196	90.35
**Social media use**					
	Ever	1259	6.92	1223	13.48
	Never	16,938	93.08	7848	86.52
**App use**					
	Ever	2358	12.96	2299	25.34
	Never	15,839	87.04	6772	74.66
**eHealth use^d^**					
	Ever	9259	50.88	9071	100
	Never	8938	49.12	0	0
**Decision to visit a physician**					
	Ever	—^e^	—	2075	22.88
	Never	—	—	6996	77.12
**Decision not to visit a physician**					
	Ever	—	—	2093	23.07
	Never	—	—	6978	76.93

^a^The value is mean age.

^b^The value is standard deviation of age.

^c^One US dollar is approximately 9 Norwegian kr.

^d^It includes the use of one or more tools, such as Web search engines, health apps, video search engines, and social media.

^e^Not applicable.

Among our subcohort of 9071 participants who sought health information using one or more eHealth tools, 2075 (22.88%) decided to visit a physician and 2093 (23.07%) decided not to visit a physician according to the information they found. Among these participants, 1047 (11.54%) decided both to visit a physician and not to visit a physician in the last year. In other words, 34.40% (3121/9071) of the subcohort used the health information as a basis for one or more decisions.

### Actual Physician Visit (n=18,197)

The independent variables selected in the multivariable logistic regression model for a physician visit were age, gender, education, household income, occupation, disease, self-reported health, Web search engine use, video search engine use, social media use, and health app use. The interactions between gender and health app use, age and disease, and age and Web search engine use were statistically significant in the multivariable model. The likelihood ratio test (χ^2^_3_=31.89, *P*<.001) of the models with and without the interaction terms indicated that the interactions had statistically significant contributions to the model. The Hosmer-Lemeshow goodness-of-fit test (χ^2^_8_=12.57, *P*=.13) indicated that the model fitted with the data very well, which means the observed and predicted values had no statistically significant difference (*P*=.13).

As shown in Table 2, the model revealed that a physician visit was predicted by Web search engine use, age, gender, education, household income, occupation, and self-reported health. Health app use, video search engine use, and social media use did not predict a physician visit.

**Table 2:** Odds ratios and 95% confidence intervals in the logistic regression analysis of the association between online health information seeking and a physician visit (n=18,197).

Variable		OR (95% CI)		*P* value	
Age (per 10-year interval)		1.27 (1.17-1.37)		<.001	
**Gender**					
	Female		1.00		
	Male		0.60 (0.55-0.66)		<.001
**Education**					
	Primary and secondary		1.00		
	Upper secondary		1.02 (0.90-1.15)		.78
	Less than 4 years of college		0.98 (0.85-1.12)		.75
	Four years or more of college		0.78 (0.68-0.88)		<.001
**Household income (US)^a^**					
	<39 000 US		1.00		
	39 000 – 61 000 US		1.28 (1.08-1.52)		.005
	61 000 – 83 000 US		1.32 (1.10-1.57)		.002
	>83 000 US		1.23 (1.04-1.46)		.02
**Occupation**					
	Full-time work		1.00		
	Part-time work		1.26 (1.06-1.50)		.008
	Unemployed		0.76 (0.49-1.20)		.23
	Housekeeping		0.59 (0.36-1.02)		.047
	Retired		1.00 (0.85-1.19)		.96
	Student/military service		0.68 (0.37-1.31)		.22
	Disability and other family welfare benefits		1.65 (1.34-2.05)		<.001
**Web search engine use**					
	Never		1.00		
	Ever		3.69 (2.33-5.83)		<.001
**Video search engine use**					
	Never		1.00		
	Ever		0.95 (0.77-1.17)		.63
**Social media use**					
	Never		1.00		
	Ever		0.89 (0.75-1.08)		.23
**App use**					
	Never		1.00		
	Ever		1.00 (0.84-1.20)		.98
**Disease**					
	Never		1.00		
	Ever		1.22 (0.79-1.88)		.38
**Self-reported health**					
	Very bad		1.00		
	Bad		0.36 (0.02-1.73)		.32
	Neither bad nor good		0.20 (0.01-0.92)		.11
	Good		0.12 (0.01-0.57)		.04
	Very good		0.07 (0.004-0.33)		.009
Gender, male; app use, ever		1.34 (1.05-1.71)		.02	
Age; disease, ever		1.16 (1.07-1.25)		<.001	
Age; Web search engine use, ever		0.86 (0.79-0.93)		<.001	

^a^One US dollar is approximately 9 Norwegian kr.

The statistically significant interaction between Web search engine use and age indicated that the association between Web search engine use and physician visit was moderated by age. In other words, the OR for Web search engine use was not constant over different ages. Therefore, the meaningful ORs of Web search engine use at different ages needed to be derived from the coefficients of Web search engine use, age, and the interaction term (see [49] for an elaborate description on how the ORs are derived). In general, Web search engine use had lower odds of a physician visit as age increased. For example, at age 40 years, those who used Web search engines had 1.99 (95% CI 1.94-2.02) times higher odds of a physician visit as compared with those who did not use Web search engines. However, at age 80 years, those who used Web search engines had 1.07 (95% CI 1.03-1.12) times higher odds of a physician visit.

Men had lower odds of a physician visit as compared with women. High household income was positively associated with higher odds of a physician visit as compared with household income less than 350,000 Norwegian kroner.

Those with 4 or more years of college education had 22% (95% CI 0.68-0.88) lower odds of a physician visit as compared with those who had a primary or secondary education. However, no difference regarding a physician visit was found for the other education levels.

A physician visit was both positively and negatively predicted depending on the type of occupation. It was positively predicted by part-time work (OR 1.26, 95% CI 1.06-1.50) and disability and other family welfare benefits (OR 1.65, 95% CI 1.34-2.05) as compared with full-time work. Those who listed housekeeping as their occupation had 41% (95% CI 0.36-1.02) lower odds of a physician visit.

### Decision to Visit a Physician (n=9071)

The independent variables selected in the multivariable logistic regression model for the decision to visit a physician were age, gender, education, occupation, self-reported health, Web search engine use, video search engine use, social media use, and health app use. The interaction between gender and social media use was statistically significant in the multivariable model. The likelihood ratio test (χ^2^_1_=4.96, *P*=.02) of the models with and without the interaction term indicated that the interaction had a statistically significant contribution to the model. The Hosmer-Lemeshow goodness-of-fit test (χ^2^_8_=5.2, *P*=.74) showed that the model’s prediction of whether the participants decided to visit a physician according to the health information they read did not significantly differ (*P*=.74) from the actual values reported by the participants.

As shown in Table 3, the model revealed that the decision to visit a physician was predicted by Web search engine use, health app use, video search engine use, social media use, age, gender, education, and occupation.

**Table 3:** Odds ratios and 95% confidence intervals in the logistic regression analysis of the association between online health information seeking and the decision to visit a physician (n=9071).

Variable		OR (95% CI)	*P* value
Age (per 10-year interval)		0.73 (0.68-0.79)	<.001
**Gender**			
	Female	1.00	
	Male	0.87 (0.78-0.98)	.02
**Education**			
	Primary and secondary	1.00	
	Upper secondary	1.18 (0.97-1.43)	.11
	Less than 4 years of college	1.25 (1.03-1.53)	.03
	Four years or more of college	1.34 (1.11-1.62)	.003
**Occupation**			
	Full-time work	1.00	
	Part-time work	0.92 (0.76-1.10)	.36
	Unemployed	0.83 (0.45-1.45)	.54
	Housekeeping	0.95 (0.35-2.26)	.92
	Retired	1.11 (0.87-1.42)	.40
	Student/military service	0.86 (0.40-1.73)	.69
	Disability and other family welfare benefits	1.39 (1.14-1.68)	<.001
**Web search engine use**			
	Never	1.00	
	Ever	2.95 (2.03-4.46)	<.001
**Video search engine use**			
	Never	1.00	
	Ever	1.43 (1.21-1.70)	<.001
**Social media use**			
	Never	1.00	
	Ever	1.43 (1.20-1.69)	<.001
**App use**			
	Never	1.00	
	Ever	1.26 (1.13-1.42)	<.001
**Self-reported health**			
	Very bad	1.00	
	Bad	1.43 (0.58-4.02)	.46
	Neither bad nor good	1.45 (0.60-4.04)	.44
	Good	1.27 (0.53-3.53)	.62
	Very good	0.96 (0.40-2.70)	.94
Gender, male; social media use, ever		0.70 (0.51-0.96)	.03

Those who used Web search engines had 2.95 times higher odds of deciding to visit a physician as compared with those who did not use Web search engines (95% CI 2.03-4.46). The association of the decision to visit a physician with Web search engine use was higher than the association with other eHealth tools. Those who used health apps had 1.26 times higher odds (95% CI 1.13-1.42) and those who used video search engines had 1.43 times higher odds (95% CI 1.21-1.70) of deciding to visit a physician.

The statistically significant interaction between social media use and gender indicated that the association between the decision to visit a physician and social media use was moderated by gender. In other words, the OR of social media use was not the same for male and female participants. Therefore, the ORs of social media use for male and female participants needed to be derived from the coefficients of social media use, gender, and the interaction term (see [49] for an elaborate description on how ORs are derived). Women who used social media had 1.42 times higher odds of deciding to visit a physician as compared with those who did not use social media (95% CI 1.31-1.55), whereas among men, there was no difference between those who used social media and those who did not use social media.

Similarly, because of the interaction, the meaningful ORs for gender were different between a social media user and nonuser, and they needed to be derived from the coefficients of social media use, gender, and the interaction term. Men who used social media had 39% (95% CI 0.55-0.69) lower odds of deciding to visit a physician as compared with women who used social media, whereas men who did not use social media had 13% (95% CI 0.86-0.88) lower odds as compared with women. A 10-year age increment was associated with 27% (95% CI 0.68-0.79) lower odds of deciding to visit a physician according to the health information found on the internet.

Higher education positively predicted a physician visit. Participants with less than 4 years of college education had 1.25 (95% CI 1.03-1.53) times higher odds of deciding to visit a physician as compared with those having a primary or secondary education, whereas participants with more than 4 years of college education had 1.34 (95% CI 1.11-1.62) times higher odds. Those who received disability and other family welfare benefits had 1.39 (95% CI 1.14-1.68) times higher odds of deciding to visit a physician as compared with full-time workers.

### Decision not to Visit a Physician (n=9071)

The independent variables selected in the multivariable logistic regression model for the decision not to visit a physician were age, gender, education, disease, Web search engine use, video search engine use, social media use, and health app use. No statistically significant interactions among the independent variables were found. The Hosmer-Lemeshow goodness-of-fit test (χ^2^_8_=6.74, *P*=.57) indicated that the model predicted the participants’ decisions not to visit a physician very well.

As shown in Table 4, the model revealed that a physician visit was predicted by Web search engine use, health app use, video search engine use, social media use, age, gender, education, and disease.

Those who used Web search engines had 2.78 times higher odds of deciding not to visit a physician as compared with those who did not use Web search engines (95% CI 1.92-4.18). The association of the decision not to visit a physician with Web search engine use was stronger than the association with other eHealth tools. Those who used health apps had 1.20 times higher odds (95% CI 1.07-1.35), those who used video search engines had 1.27 times higher odds (95% CI 1.07-1.51), and those who used social media had 1.28 times higher odds (95% CI 1.10-1.49) of deciding not to visit a physician.

A 10-year age increment was associated with 22% (95% CI 0.73-0.83) lower odds of deciding not to visit a physician according to the health information found on the internet. Men had 33% lower odds of deciding not to visit a physician as compared with women (95% CI 0.60-0.74).

**Table 4:** Odds ratios and 95% confidence intervals in the logistic regression analysis of the association between online health information seeking and the decision not to visit a physician (n=9071).

Variable		OR (95% CI)	*P* value
Age (per 10-year interval)		0.78 (0.73-0.83)	<.001
**Gender**			
	Female	1.00	
	Male	0.67 (0.60-0.74)	<.001
**Education**			
	Primary and secondary	1.00	
	Upper secondary	1.09 (0.90-1.33)	.37
	Less than 4 years of college	1.29 (1.07-1.56)	.01
	Four years or more of college	1.20 (0.99-1.44)	.06
**Web search engine use**			
	Never	1.00	
	Ever	2.78 (1.92-4.18)	<.001
**Video search engine use**			
	Never	1.00	
	Ever	1.27 (1.07-1.51)	.006
**Social media use**			
	Never	1.00	
	Ever	1.28 (1.10-1.49)	.001
**App use**			
	Never	1.00	
	Ever	1.20 (1.07-1.35)	.002
**Disease**			
	No	1.00	
	Yes	1.18 (1.05-1.32)	.006

The decision not to visit a physician was positively predicted by education. Participants with less than 4 years of college education had 1.29 times higher odds of deciding not to visit a physician as compared with those having a primary or secondary education (95% CI 1.07-1.56). Those who had one or more diseases in the past year had 1.18 times higher odds of deciding not to visit a physician as compared with those who did not have a disease (95% CI 1.05-1.32).

## Discussion

### Actual Physician Visit

This study examined the association between a physician visit and health information seeking using eHealth tools (ie, Web search engines, video search engines, social media, and health apps). Our results indicated that a physician visit was positively predicted by health information seeking on Web search engines, which confirms the findings of some prior studies [37-40]. The use of Web search engines had a stronger association with a physician visit possibly because online health information seeking often starts with a Web search engine (ie, Google) [17,40]. However, the association was moderated by age, where the OR decreased as age increased. A physician visit was also positively predicted by higher household income, female gender, and older age, which is in line with the results found in other studies [37,39,50]. A physician visit was both positively and negatively predicted depending on the type of occupation.

A previous study has shown that the difference in physician visits between highly educated and less educated individuals is gradually decreasing in Norway [51]. A recent Norwegian study on patients with diabetes found no difference (95% CI 0.44-5.59) regarding physician visits between highly educated and less educated individuals [44]. In general, our results were in line with existing findings on the lack of a difference in physician visits between highly educated and less educated individuals. However, we found that those who had 4 or more years of college education had lower odds of physician visits as compared with those who had primary and secondary education.

### Decision to Visit a Physician

Of 9071 participants who sought health information online, 3121 (34.40%) used the information they read to decide whether they needed to visit a physician. This is lower than the number reported in previous studies [4,8,40]. The difference can possibly be attributed to the fact that the participants included in this study were older than the participants in the other studies. In addition, differences in the health care systems (ie, publicly and privately funded) of the countries could cause varying access to health care services and, consequently, influence the reasons for seeking health information on the internet. It is possible that tax-funded health systems with small or no payment from patients are conducive to the use of traditional face-to-face consultations and to a lower use of eHealth as compared with health systems that are dependent on the financial situation of individuals. A previous study found that 88% of Norwegians prefer to see their GPs face-to-face [52]. Hence, the most common reasons Norwegians seek health information via the internet could be for purposes other than to decide whether to visit a physician.

Studies have shown that people seek health information on the internet for many reasons including deciding whether to visit a physician; preparing for a physician appointment; and reassurance, second opinion, and expanding knowledge on the information received after a physician visit [15,16,18,48]. Thus, the results of our model on a physician visit provided general information on how health information seeking is associated with a physician visit. On the other hand, the models for the decisions to visit a physician and not to visit a physician provided information on how health information seeking is specifically associated with each of these decisions.

Similar studies often use a single variable that measures whether participants decide to visit a physician according to the health information they read on the internet [40]. However, our study used two variables that measure participants’ decisions to visit a physician and not to visit a physician. Our results showed that of 9071 participants who sought health information using eHealth tools, 1047 (11.54%) decided both to visit a physician and not to visit a physician in the last year, a finding that would not have been captured by a single question.

Health information obtained from eHealth tools (ie, Web search engines, health apps, video search engines, and social media) positively predicted both the decision to visit a physician and the decision not to visit a physician. The positive associations of eHealth tools with both decisions would not have been revealed with a single model for both decisions. The association between social media use and the decision to visit a physician was moderated by gender. The association of Web search engine use with both decisions was stronger than the associations of other eHealth tools, which may be explained by the fact that online health information seeking often starts with a Web search engine (ie, Google) [17,40].

Each eHealth tool showed a slightly stronger association with the decision to visit a physician than with the decision not to visit a physician. In other words, the odds of the decision to visit a physician according to the health information read online was greater than the odds of the decision not to visit a physician. Therefore, health information seeking via the internet overall slightly increased physician visits, supporting our findings on the positive association between health information seeking via Web search engines and actual physician visits.

Women had higher odds of making a decision according to the health information they read on the internet as compared with men. Receiving disability and other family welfare benefits was also associated with higher odds of deciding to visit a physician as compared with full-time workers. This difference might be attributed to the disease conditions that led to the welfare benefit.

Participants with one or more diseases had higher odds of deciding not to visit a physician according to the health information they read on the internet as compared with those who did not have a disease. Difficulty to interpret medical knowledge found on the internet is considered to increase physician visits [37]. Another study revealed that individuals diagnosed with a medical condition had a positive association with higher knowledge from the health information they read as compared with those who were not diagnosed with a disease [34], which is probably because they were better equipped to understand the health information. Consequently, these participants were able to make a decision according to what they read. These results highlight the potential of eHealth for managing chronic conditions.

Being older and less educated were associated with lower odds of deciding to visit a physician and deciding not to visit a physician according to the health information found on the internet. In other words, older and less educated individuals had lower odds of using the health information they found online to make a decision. These characteristics are known to negatively predict the use of eHealth in general [17,40,45,53]. These participants’ lower odds of decision-making according to online health information might be attributed to a lack of necessary skills for navigating the internet, doubts regarding the quality or relevance of the health information available on the internet, or a view that physicians know best [17,45]. These differences signal a need to provide educational support to these population groups in particular.

Many individuals are likely to search the internet for health information to prepare for a physician appointment that has already been decided [8]. Deciding to visit a physician or not to visit a physician according to online health information is a choice individuals may make in connection with each search. There can be many rational reasons to see a physician after finding health information online. For instance, the information can substantiate worries that people may have concerning symptoms or illnesses. In some cases, people can find information suggesting that they are at increased risk of having or developing some illnesses, such as cardiovascular disorders and diabetes [54]. Some disorders can also be screened or diagnosed by the patient with the help of information found online, and this may necessitate further evaluation and treatment by health professionals [55].

However, we also recognize that the decision to visit or not to visit a physician after obtaining online health information is multifactorial. The findings of this study can be understood in light of the theory by Kuhltau [31,32] regarding information searching. The Information Search Process Model describes information seeking in six phases. It sees the process as complex and takes into consideration both cognitive and emotional factors, recognizing that information searchers can experience a range of feelings throughout the process, including optimism, satisfaction, confusion, and disappointment.

In this study, overall, we found that online health information is associated with increased use of traditional health services (ie, visiting a physician). By drawing on the Information Search Process Model [31,32], we understand that simply finding the relevant information may not be the endpoint of the process and that many online health information searchers will have feelings, such as confusion and frustration, even after finding the information they were looking for online. The decision to visit a physician following online health information searching may therefore also, in part, be based on emotional factors. People with health anxiety are more likely to visit a physician after finding health information online [33], supporting the idea that emotions also play an important role in the decision-making process.

### Implications

We examined the importance of some central factors, such as demographic characteristics and health status, in the association between online health information searching and the decision to visit or not to visit a physician. However, drawing on the theory about health information searching [28-32], we emphasized the complexity of our topic, pointing out that the decision to visit a physician or not to visit a physician is based on a range of factors that we lack information about, including cognitive factors and emotional factors.

We believe that online health information has its place as part of a well-functioning health service and that much can be done to further improve the availability of quality online health information that will benefit patients and the general public [56,57]. Although we believe that this study has made a great contribution to the literature, many questions remain, and they should be addressed in future studies.

### Future Research

There is a need for more studies related to this topic. For instance, studies that include psychological data about participants could provide important insights into the relevance of cognitive and emotional factors regarding how health information searchers react with respect to seeing a physician following health information searching [33]. Further studies are needed to examine what aspects of information sources contribute to deciding either to visit a physician or not to visit a physician. Issues, such as emotional content, understandability, usability, and readability, could be of importance [56]. Future studies could also address how health information searching influences other health-related behaviors, such as posting about health and illness on social media. Studies combining data on health information seeking via the internet and outcomes of physician visits are also needed to investigate whether access to eHealth might be conducive to unnecessary physician visits.

### Strengths and Limitations

This study is based on cross-sectional data, with a large sample size and relatively good response rate. The invitation for participation was sent to all inhabitants of Tromsø aged 40 years or above by mail, which contributes to the representativeness of the study sample. Although prior studies have shown that the reproducibility and validity of self-reported findings from the Tromsø Study are quite high [58,59], there is still a possibility of recall bias, which may affect the validity of the results. High proportions of nonrespondents were men and older individuals, and people with one or more diseases and those who had physician visits were overrepresented among the participants [60].

Both the decision to search for health information on the internet and the decision to visit a physician or not to visit a physician are likely to be influenced by a range of different factors, including cognitive factors and emotional factors. Similarly, how people react to the use of eHealth services and traditional face-to-face services will be influenced by many different factors. We have taken some of the factors, such as demographic factors and health status, into account in this study regarding the relationship between online health information searching and physician visits, but there was a lack of information regarding many other variables that might be of interest. For instance, there was a lack of data on health anxiety that may moderate the relationship between health information seeking and the decision of whether to visit a physician.

The cross-sectional study design did not make it possible to establish causality, and the results might be affected by unmeasured confounding variables. With the current widespread use of the internet on smartphones and tablets, the difference between the use of search engines and apps may be blurry for some participants, which may affect the results.

### Conclusions

In this study, we examined the association between health information seeking on the internet with a physician visit, making a decision to visit a physician, and making a decision not to visit a physician. We found that both the decision to visit a physician and the decision not to visit a physician were positively predicted by searching for health information online. However, searching for health information on the internet was associated with a resultant increase in physician visits. The implication of this finding is that for our participants, overall, online health information did not replace or reduce the need for traditional face-to-face health services. As such, online health information does not, at least in our study, stand out as a means for saving resources or resolving the demands on traditional resource-strained health services.

## Abbreviations

eHealth: electronic health
GP: general practitioner
Tromsø 7: the seventh survey of the Tromsø Study
